# Children’s Perspectives on Outpatient Physician Visits: Capturing a Missing Voice in Patient-Centered Care

**DOI:** 10.3390/children8010034

**Published:** 2021-01-07

**Authors:** Jessica S. Dalley, Barbara A. Morrongiello, C. Meghan McMurtry

**Affiliations:** 1 Department of Psychology, University of Guelph, Guelph, ON N1G 2W1, Canada; jdalley@uoguelph.ca (J.S.D.); bmorrong@uoguelph.ca (B.A.M.); 2 McMaster Children’s Hospital, Hamilton, ON L8N 3Z5, Canada; 3 Department of Paediatrics, Western University, London, ON N6A 3K7, Canada; 4 Children’s Health Research Institute, London, ON N6C 2V5, Canada

**Keywords:** physician, primary care, children, patient participation, patient-centered care

## Abstract

Actively involving children in their healthcare is a core value of patient-centered care. This is the first study to directly obtain children’s detailed perspectives on positive and negative aspects of outpatient physician visits in a primary care setting (e.g., checkups) and their preferred level of participation. Individual interviews were conducted with 167 children (female *n* = 82, male *n* = 85; ages 7–10, M_age_ = 8.07 years, SD = 0.82). Open-ended questions were used so that children’s responses were not confined to researchers’ assumptions, followed by close-ended questions to meet specific objectives. Quantitative content analysis, correlations, logistic regression, and Cochran’s Q were used to explore the data. Children were highly fearful of needle procedures (61%), blood draws (73%), pain (45%), and the unknown (21%). Children indicated that they liked receiving rewards (32%) and improving their health (16%). Children who were more fearful during physician visits wanted more preparatory information (ExpB = 1.05, Waldx^2^(1) = 9.11, *p* = 0.003, McFadden’s R^2^_2_ = 0.07) and more participation during the visit (ExpB = 1.04, Waldx^2^(1) = 5.88, *p* = 0.015, McFadden’s R^2^_2_ = 0.03). Our results can inform efforts to promote positive physician visit experiences for children, reduce procedural distress, and foster children’s ability to take an active role in managing their health.

## 1. Introduction

Children rely on outpatient visits to family physicians, pediatricians, or general practitioners (herein “physician”) for regular preventative health assessments (e.g., well-child visits, vaccinations) and treatment of acute illness or injury [1,2,3]. In Canada, these visits occur in non-hospital settings and are more frequent than hospital visits for most Canadian children [1,2,3]; despite this, most research regarding patient perspectives of pediatric medical services is specific to hospitalization, rather than visits to a physician, e.g., [4,5,6]. Although there are studies eliciting children’s perspectives on isolated needle procedures [7,8,9,10] and hospitalization [5,11,12,13,14,15,16], there are two major gaps in the literature: (1) children’s perspectives on positive and negative aspects of physician visits have not been obtained; and (2) no research can speak to children’s preferences for participation during physician visits. Understanding children’s perspectives on physician visits could lead to significant improvements in their healthcare; the identification of both positive and negative aspects could elucidate intervention targets. However, research regarding children’s medical care typically relies on the opinions of clinicians and parents, rather than children themselves [4,5,12]. This is problematic because parents and clinicians may not accurately predict children’s evaluations, and children should also be included as stakeholders in research regarding their healthcare [16,17,18,19,20,21,22].

Actively involving children in healthcare research and their own treatment is a core value of ethical, patient-centered healthcare [18,19,20,21,22]. Children’s participation refers to their receipt of developmentally appropriate procedural information and allowing them to ask questions and share opinions [12,13,16,19,20,21,22]. Guidelines created by Hart (1992) for UNICEF promote a “ladder of participation” for children”, with four levels of increasing participation based on the child’s competence and cognitive development, as follows: (1) simply providing information about medical procedures and the child’s health; (2) listening to children’s views; (3) taking their views into account to make decisions; and (4) allowing competent adolescents to be the primary decision-maker for their healthcare. Hart’s ladder of participation demonstrates that children can and should always be active participants in their healthcare; of course, children’s degree of participation will vary based on their level of cognitive development, the parent’s preferences, and the children’s own preferences for their participation. Children’s participation in their care during physician visits may help to increase their understanding of health concepts, which in turn acts as preparation for making more serious medical decisions in the future [23,24]. As quoted by Lewis et al. (1991), *“The relatively passive role for children* [in the context of medical settings] *contrasts with the efforts to promote knowledge, healthy habits, and a sense of control over health that are at the heart of many recent health education interventions for children”* (p. 351) [21]. Parents and healthcare providers need to truly understand the perspective of children during physician visits, as this will inform the provision of patient-centered care, interventions to promote children’s development of healthcare knowledge, and their ability to take an active role in managing their health.

Children’s ability to understand medical concepts and communicate their needs and preferences will be dependent, in part, on their developmental stage. It is well documented that children as young as five years old prefer to be provided information regarding their medical care and report insufficient preparatory information regarding procedures [5,14,20,25]. This lack of preparatory information can result in exacerbated pain and distress, increased procedure and recovery time, and avoidance of medical care in adulthood [5,7,8,9,14,15,16,25,26]. However, hospitalized children aged 7–14 reported that they felt little control over the procedures performed on them during hospitalization and that parents and healthcare providers inhibited their efforts to participate in their own healthcare, resulting in increased feelings of powerless [5,25]. Further, children have reported that adults disregard their signals for distress, and when they directly ask for help in managing fear and pain, they feel ignored [25]. Certainly, healthcare providers and parents want to be helpful to children; therefore, understanding children’s own perspectives can help to inform adults on how to optimally manage children’s needs during medical procedures. While studies eliciting children’s perspectives on their own healthcare have increased in recent years, e.g., [5,6,14,25], research regarding children’s participation in decision-making in the context of physician visits is scarce.

In addition to distress related to the unknown, lack of preparatory information, and difficulties having their perspective heard by adults, the extant literature has demonstrated children have fears about specific medical procedures. Firstly, it is well established children are fearful of needles and pain [17,27,28,29,30]; this is particularly important to understand because vaccinations typically occur at physician visits, potentially leading to avoidance or increased distress. This fear is likely connected in part to children’s belief that they will not be informed of upcoming procedures [5] and not have their pain and distress adequately managed [30,31,32]. Even non-painful procedures classified as minor by healthcare professionals and parents may be considered fear-inducing by some children. For example, a small subset of elementary school children reported being fearful of purportedly minor procedures (e.g., throat cultures, ear checks, and heart checks) in two studies regarding children’s fears [29,33]. Currently, there is limited knowledge of what specific aspects of physician visits children find fear-inducing, as existing studies regarding children’s medical fears have been conducted in the context of general fears (including non-medical fears; [34,35]), or in the context of hospitalization, e.g., [15,25,36], with few exceptions [29,33]. Information about children’s fears is particularly important in order to understand the aspects of the medical experience for which children require intervention to manage their distress, as procedural fear experienced in childhood can relate to procedural pain and poor coping in adulthood, as well as medical avoidance [8,26].

Lastly, the current study is the first to elicit children’s own perspectives on what they enjoy about physician visits. Research regarding positive aspects of physicians is scarce, but understanding the aspects of physician visits that children enjoy is essential for the improvement of children’s healthcare. It is likely that children feel both positive and negative emotions during medical experiences; for example, hospitalized children have reported feeling both fearful of procedures as well as taking pride in facing their fears [25]. Many hospitalized children aged 6–17 have reported positive experiences during hospitalizations, including positive relationships with health professionals (particularly nurses), taking pride in facing their fears, and advantages such as watching TV and missing school, e.g., [5,6,37]. Lastly, children identified the hospital as a place in which they can be healed and recover [6]. It is clear that children feel positively about certain aspects of their medical experiences. However, the aforementioned studies involved hospitalized children, and research is needed to determine which aspects of physician visits children view positively. Through understanding what children enjoy about physician visits, interventions to promote these positive aspects can be developed.

### Objectives and Hypothesis

This study is the first to interview children directly to gain a detailed understanding of their perspectives on outpatient physician visits. This information can be used to inform interventions designed to increase children’s health knowledge, correct children’s misperceptions, and/or promote children’s participation in their own healthcare. The specific objectives and hypotheses of the proposed study are described below:

1. The first objective was to determine what aspects of physician visits children dislike, including aspects that they report as aversive and fear-inducing. Specifically, it was hypothesized that children would be fearful of aspects of visits that include pain, needle procedures, separation from their family, and the unknown (Hypothesis 1: H_1_).

2. The second objective was to determine what aspects of physician visits children find positive. While there is some research regarding children’s positive medical experiences, there was not enough strong evidence to formulate an evidence-based prediction for this objective.

3. The third objective was to explore children’s perspectives of the information provided to them by doctors during physician visits. It was hypothesized that children would report that they received inadequate preparatory information from doctors (H_2_). Additionally, exploratory sub-analyses regarding the relations between children’s age and fear and their desire for more preparatory information were conducted.

4. The fourth objective was to determine children’s perspectives of their participation during physician visits. It was hypothesized that children would report believing that they do not have a choice in what happens during physician visits (H_3_) and would prefer more control over the procedures that occur (H_4_). Additionally, exploratory sub-analyses regarding the relations between children’s age and fear on their desire for increased participation during physician visits were conducted.

## 2. Materials and Methods

### 2.1. Participants

Children in grades 2–4 were recruited by letter from a convenience sample of four schools in the Waterloo Region (ON, Canada) from January to June 2015. Children this age can identify common medical objects, are likely to have had several memorable experiences with a physician to report, and have the cognitive and language capabilities to answer questions [5,6,14,16,20,25,38]. One hundred and seventy-eight parents gave consent (41% response rate), consistent with previous school-based studies requiring active consent [39]. Ninety-three percent of children with parental consent gave assent, leading to a final sample of 167 seven to ten-year-olds (M_age_ = 8.07, SD = 0.82), including 82 females (49%) and 85 males. A sample size of 167 is sufficient to detect medium-sized effects at a power of 0.80 and an alpha of 0.01 for correlations, chi square (up to 2 degrees of freedom), and multiple regressions with up to 5 predictors.

### 2.2. Methods

The university and school board Research Ethics Boards granted approval (#14OC042). Data were collected through 15–20-min individual interviews conducted at school by two female undergraduate research assistants (RA) and the first author (graduate student in clinical psychology). Researchers were trained in ethical interviewing techniques with vulnerable populations. The RAs were trained on the interview through demonstration by and practice with the first author. Participants were informed that the purpose of the interview was to learn more about children’s experiences regarding doctor’s visits. The researcher read questions aloud and recorded the child’s answers verbatim.

### 2.3. Interview Materials

The structured interview is available from the corresponding author. Children’s age, sex, grade, and school were recorded. It is considered best practice to use a combination of open- and close-ended questions to ascertain children’s self-reported fear [29,34,35,36]. Further, given that there was little past research to guide the creation of interview topics, it was important that open-ended questions be used so that children’s responses were not confined based on assumptions made by the researchers. Open-ended questions regarding fears were followed by targeted questions in order to meet study objectives.

Specifically, children were asked a series of open-ended questions first, followed by closed-ended questions on the same topics, including: positive experiences, rewards after visits, fears of specific equipment and procedures (with the aid of pictures), pain, being uninformed about medical procedures, separation from parents, and contracting germs. These questions were adapted from existing questionnaires and other medical fear research [29,33,34,35,36,40,41,42]. For the closed-ended questions, the Children’s Fear Scale (CFS) [41] was used to gather participants’ level of fear for a given procedure/situation, with scores ranging from 0 to 4 (0 = no fear, 4 = high fear); the scale has evidence of construct validity as well as test–retest and inter-rater reliability in capturing procedure-related fear in this age group. Seriation tasks have been effective in screening school-age children’s ability to use faces scales to self-report levels of procedural pain; therefore, we inferred that a seriation task would be a useful tool for screening whether children could use a faces scale to self-report fear. Thus, before answering questions regarding their fears, children were asked to place the five faces of the CFS in order of increasing intensity. If children could not place the five faces used in the CFS in order, they were asked to complete a seriation task with five measuring cups of increasing size and answered questions regarding their fears with these measuring cups. Eight of 167 participants (4% of participants) were not able to place the five faces in order; all eight of these participants were able to place the cups in order, so these were used to indicate the magnitude of their fear.

There is a need for caution in designing, analyzing, and interpreting data from studies involving younger children. The sample within this study was aged seven through ten, which is consistent with the extant literature that investigates children’s fears [42] and experiences with hospitals or needle procedures, e.g., [5,12,13,14,25,33]. The structured interview was also piloted with ten children within this age range.

### 2.4. Coding Process

Data obtained through open-ended questions were analyzed using content analysis, which is an objective and systematic approach for describing qualitative data in a quantitative manner by obtaining the frequency at which specific content emerges from the data [43,44,45]. First, Coders 1 (first author) and 2 (corresponding author) developed an initial coding manual using a combined inductive (based on initial reading of responses) and deductive (extant literature medical fear, pediatric pain, patient-centered care) approach. Next, Coders 1 and 3 (undergraduate RA) coded a random sample of responses from the present data using the manual; following a test for inter-rater reliability and discussion between coders, problematic or unclear coding criteria were modified and the manual was finalized. Coders 1 and 3 then coded the entire dataset using the refined coding manual; all subcategories had a mean inter-rater reliability of at least Cohen’s κ = 0.80, acceptable according to Altman’s Kappa Benchmark Scale [45]. This double coding of all of the data was used to enhance the trustworthiness of the data [44]. Disagreements between Coders 1 and 3 were resolved by Coder 2. All coders were from psychology backgrounds with an emphasis on training in pain and child development.

### 2.5. Analytic Plan

All data collected were entered into Statistical Package for the Social Sciences (SPSS), version 22 (IBM Corp., Armonk, NY, USA) and then checked for accuracy by two undergraduate research assistants. Participants were asked both closed- and open-ended questions; both qualitative and quantitative analyses were used. Analyses are described below by type.

#### 2.5.1. Descriptive Analyses

Frequencies for children’s fear ratings for each item on the closed-ended fear questions were calculated. Frequency analyses were also completed to explore children’s responses to open-ended questions regarding their spontaneously reported fears; aspects of physician visits that children enjoy; content of preparatory information that children prefer; who makes choices for children during physician visits; and aspects of physician visits in which children want more of a choice. Next, children’s indications of whether or not they receive preparatory information from their doctor, and whether or not they want more preparatory information, were analyzed through frequency analyses. Lastly, children’s indications of whether or not they have a choice during physician visits, and whether or not they want more of a choice, were analyzed through frequency analyses.

#### 2.5.2. Cochran’s Q Analyses

Cochran’s Q analyses were used to determine the differences in proportions for selected coding categories within children’s responses; these were selected post-hoc and adjusted through the Bonferroni correction (α = 0.05/# of comparisons in the Cochran’s Q analysis). Effect sizes for Cochran’s Q analyses were calculated using Serlin, Carr, and Marascuilo’s (1982 [46]) formula for eta squared, where 0.01 is a small effect, 0.06 is a medium effect, and 0.13 is a large effect [47].

#### 2.5.3. Pearson Correlations

Pearson correlations were calculated to determine the relation of children’s age and their fear of (1) needles, (2) pain, (3) the unknown, and (4) separation from parents. Pearson correlations were used, given that children’s age and fear were continuous variables and assumptions for linearity and normality were met. Correlations are considered small if below 0.30, medium if between 0.30 and 0.50, and large if above 0.50 [47].

#### 2.5.4. Multinomial Logistic Regressions

A forced-entry, multinomial logistic regression was conducted to predict children’s preference for more preparatory information during physician visits from their age and total fear score. Another forced-entry, multinomial logistic regression was conducted to predict children’s preference for more choice in what occurs during physician visits from their age and total fear score. Children’s total fear score was derived by summing each participant’s ratings of fear in response to the closed-ended fear questions (minimum score 0, maximum score 64; higher score indicates higher total fear ratings). Effect sizes for regressions were calculated using McFadden’s pseudo-R squared, where effect sizes below 0.10 are considered to be small [48,49].

## 3. Results

In the following, “spontaneous” responses denote answers/data given in response to open-ended questions; Table 1, Table 2 and Table 3 contain these data and illustrative examples of codes.

### 3.1. Children’s Negative Experiences

**Figure 1:**
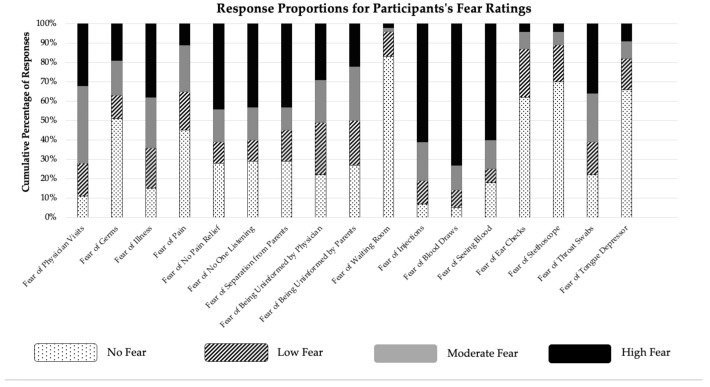
Response proportions (%) for children’s fear ratings using the Children’s Fear Scale (McMurtry et al., 2011) [41] in response to closed-ended questions in the interview.

Table 1 presents the frequencies and illustrative examples for each category of responses to the open-ended questions on fears and negative experiences. Figure 1 shows frequencies in response to closed-ended questions using the CFS about fear of specific aspects of physician visits. The proportion of responses spontaneously reporting needles as a feared experience (57%) during physician visits was significantly higher than the proportion indicating a fear of pain (13%; χ^2^(1) = 59.88, *p* < *0*.001, η^2^_Q_ = 0.05), fear of the unknown (12%; χ^2^ (1) = 55.69, *p* < *0*.001, η^2^_Q_ = 0.05), and fear of other medical procedures (21%; χ^2^(1) = 38.30, *p* < *0*.001, η^2^_Q_ = 0.03). Fear ratings for receiving medicine through a needle decreased with age (*r* = −0.226, *p* = 0.002), as did fear of blood draws (*r* = −0.214, *p* = *0*.003). Fear of pain was spontaneously reported by 13% of participants. Ratings of fear of pain and fear that no one would help them if they were experiencing pain at the doctors’ both decreased with age (*r* = −0.171, *p* = 0.02, and *r* = −0.161, *p* = 0.02, respectively). Twelve percent of participants spontaneously reported fear of not knowing what will happen during physician visits. There was no difference in proportion between children’s spontaneous reports for fear of pain versus of the unknown, χ^2^(1) = 0.111, *p* = *0*.74, η^2^_Q_ = 0.006. Children’s fear of not being provided information by their doctor showed no significant relation with age (*r* = −0.082, *p* = *0*.15), whereas fear of feeling uninformed by their parents was decreased with age (*r* = −0.199, *p* = *0*.005).

### 3.2. Children’s Positive Experiences

Table 2 presents the frequencies and illustrative examples for each category of responses to the open-ended questions about positive experiences during physician visits. Participants spontaneously described enjoying the rewards that they receive after physician visits (32%), improved personal health (16%), child-friendly activities in the waiting room (14%), positive interactions with healthcare personnel (14%), and enjoying some medical procedures (13%). The proportion of children’s responses for enjoying rewards during physician visits was significantly higher than responses for other positive experiences, χ^2^ (1) = 27.36, *p* < 0.001, η^2^_Q_ = 0.03; an omnibus Cochran’s Q test did not indicate any differences among the proportions of the non-reward-related positive experiences, χ^2^ (1) = 0.896, *p* = 0.83, η^2^_Q_ = 0.005.

### 3.3. Children’s Participation

Table 3 presents frequencies and illustrative examples from each category of responses to questions regarding children’s preferences for more participation and information during physician visits. Sixty percent of participants indicated that their doctor provided them with sufficient preparatory information, whereas 36% wanted more information (4% did not answer). A forced-entry, multinomial logistic regression showed that children with higher summed fear scores wanted more preparatory information from their doctor, ExpB = 1.05, Waldx2(1) = 9.11, *p* = 0.003 (95% Confidence Interval (CI) = 1.02–1.08, McFadden’s R22 = 0.07); age was not a significant predictor. Children requested general information about what will occur during the appointment (34%), and specific information regarding upcoming needle procedures (18%) and non-needle-related medical procedures (19%). Pairwise comparisons with a Bonferroni correction (α = 0.05/3 = 0.017) revealed that children were more likely to want preparatory information regarding needle procedures versus pain (χ^2^ (1) = 18.00, *p* < 001, η^2^_Q_ = 0.05); non-needle medical procedures versus pain (χ^2^ (1) = 17.86, *p* < 0.001, η^2^_Q_ = 0.05); and their personal health than pain (χ^2^ (1) = 9.14, *p* = 0.002, η2Q = 0.03). Eight percent indicated that they would not want any preparatory information whatsoever.

Sixty percent of all participants reported that they do not have a choice in what occurs during visits. However, in a follow-up question, only 34% agreed that they would like more of a choice regarding what occurs during physician visits. A forced-entry, multinomial logistic regression showed that children’s summed fear scores predicted their preference for more choice, ExpB = 1.04, Waldx2(1) = 5.88, *p* = 0.02 (95% CI = 1.01–1.07) McFadden’s R22 = 0.03; age was not a significant predictor.

## 4. Discussion

Children’s perspectives are integral to patient-centered research and clinical practice. Children’s participation will vary based on their cognitive development, parental and child preferences, and appropriate care. Unfortunately, their perspectives are often overlooked. Our results may inform efforts to promote positive physician visit experiences for children, reduce their procedural distress, and foster children’s ability to take an active role in managing their health.

Promoting aspects of visits that children enjoy is integral to improving their physician visit experiences [5,6,13]. Children were most likely to report that they enjoyed the rewards that they received at their visit (e.g., food, small toys). Participants appreciated how visits to the physician resulted in cured illness, healed injuries, and/or confirmations that they were healthy. Consistent with hospitalized samples [5], children preferred when physicians had positive communication styles (e.g., funny, nice) and waiting room activities appropriate for children of all ages. Thus, the present results suggest that improving children’s satisfaction with physician visits could be implemented easily and at low cost.

Children described specific aspects of physician visits that they found to be fear-inducing, which can likewise provide suggestions for intervention efforts. The most commonly endorsed fear was undergoing a needle procedure, including both injections and blood draws. These fears were weakly related to age such that fears were higher for younger children. Children associate visits to the physician with needle procedures [50]; therefore, intervention to reduce anticipatory distress is necessary regardless of whether a needle procedure is actually scheduled. Children reported fear of experiencing pain without relief from clinicians, which is consistent with other research indicating inadequate management of children’s pain [26,27,51]. Children and parents would benefit from learning about pain management options available to them so they can advocate for procedural pain relief, if needed.

Children were equally likely to fear the unknown and pain in the context of physician visits. This provides further support for the supposition that fear of medical procedures extends beyond fear of experiencing pain; therefore, interventions to reduce fear should extend beyond pain management. This is consistent with results from interviews with hospitalized children showing that children’s distress is partly based on feeling uncertain about what procedures will occur [5,12,14,25]. Further discussion on the provision of information appears below. Notably, some participants were fearful of purportedly minor procedures such as throat swabs, ear checks, and having a tongue depressor put in their mouth. Most children endorsed feeling low amounts of fear for these types of procedures, which may be responsive to straightforward education strategies (e.g., books, pamphlets). When discussing throat swabs, children reported that they worried that they would choke on the throat swab, it would be painful, it would get stuck, or they would not be able to breathe. While this study was completed before the COVID-19 outbreak, it is logical to assume that children may have similar concerns about nasal swabs.

Children reported they are not involved in conversations regarding what occurs during physician visits. The exclusion of children from active healthcare participation is antithetic of widespread efforts to “*promote knowledge, healthy habits, and a sense of control over health*” [21], p. 351. Children’s participation may help to increase their understanding of health concepts, which could in turn act as preparation for future, more serious medical decisions [10,11,15,19,20,21,22,23,24]. The current data suggest that most children are content with the amount of participation that they have during physician visits. However, one third of children wanted increased participation and sought the opportunity to ask the doctor questions and share their opinions.

As expected, many children reported that their doctor does not provide them with preparatory information during their visits; yet, consistent with desire for participation, only a third of participants wanted more information. This finding is in contrast to hospitalized children and adolescents, who seem to prefer to be provided with more preparatory information regarding their medical care [5,16,25], and our own findings about fear of the unknown. The ambiguity of these findings may be explained by previous research that suggests that information provision can be effective in reducing procedural distress and pain in some instances [13,16,19,20] but can also be ineffective or harmful for some children [52]. Therefore, research is needed to determine if information provision interventions are effective for highly fearful or avoidant children in the context of physician visits. Notably, children who endorsed higher ratings of fear to the closed-ended fear questions were more likely to want increased participation and preparatory information in the current study.

The current study is the first to interview children directly regarding their experiences with outpatient physician visits. A major strength of this study was its grounding in the principles of patient-centered health research. Interviews were conducted with children directly, using developmentally appropriate methods, in order to obtain their unique perspectives on physician visits. Historically, children’s cognitive and emotional development were seen as barriers for their inclusion in research regarding improving children’s healthcare [6]. Children in the concrete-operational stage, which is the developmental stage of children in the current study, have demonstrated understanding of concepts related to health and illness in previous research, e.g., [5,6,14,16,25]. Further, the majority of children in the current study were able to provide some information regarding the provision of preparatory information and their degree of participation during physician visits. Overall, children are capable of providing some suggestions for how their healthcare can be improved, and their perspectives should be considered in combination with those of parents and healthcare professionals in efforts to improve physician visits.

The current study has several limitations that should be addressed in future research. Firstly, although our results detail aspects of physician visits that should be continued or changed, there is no guarantee that implementing these suggestions will actually result in outcomes such as reduced procedural distress and fear, increased satisfaction with healthcare services, and adherence with medical recommendations. Furthermore, research must be conducted to determine the feasibility of incorporating the findings from exploratory and intervention research into practice during physician visits. Additionally, several other variables could have been investigated to explain variability in children’s fears, preference for preparatory information, and preference for increased participation during physician visits. Based on research in other settings, it is possible that children’s trait anxiety, parents’ trait anxiety and parent–child interactions during procedures, children’s coping strategies, and children’s memories of previous medical procedures could impact their experiences at physician visits [7,16,32,41,52]. Next, a strength of the current study was its rich detail on children’s negative and positive perspectives of physician visits within a narrow developmental stage. Nevertheless, including a wider age range of children may have resulted in enhanced information regarding the impact of age specifically on children’s medical fears, as well as the ability to compare patterns across different age ranges. Further, future research may wish to compare differences in experiences between males and females. Our objective was not to investigate whether particular factors or groupings emerged among the various quantitative fear ratings, but this would be an interesting area for future research. We did, however, create a sum total of these ratings which, along with age, was used to predict children’s preference for more preparatory information and for more choice during physician visits. The use of such a total score in this manner has its limitations and should be further examined in future research. Additionally, although the majority of children were able to provide some information about increased preparation for physician visits and more choice in what happens, the number of children (n = 40; 24%) who provided no response to these questions was twofold higher than for any of the other questions. Lastly, it would be beneficial to interview children directly to obtain their perspectives on their experience of primary care during the COVID-19 pandemic, including their fears related to COVID-19 nasal swabs, getting diagnosed with COVID-19, and attending a primary care appointment during the pandemic. Children in the current study reported fear of the unknown and preferring to learn more about their health; therefore, it would be interesting to understand children’s perspectives on managing their health and their preference for preparatory information during an unpredictable health crisis.

Physician visits are common for most children throughout childhood (e.g., well-child visits, primary care). Visits to the physician are a complex, multi-faceted experience for children, involving both positive and negative aspects. Children clearly communicated their perspectives on physician visits and should be valued as key stakeholders in research regarding their healthcare. The current findings can inform the development of patient-centred care programs to improve children’s experiences of physician visits and foster their ability to take an active role in managing their health as they develop.

## Figures and Tables

**Table 1:** Frequencies and illustrative examples from each category of responses to questions regarding children’s (*n* = 167) fears and negative experiences during physician visits. The categories are not mutually exclusive, meaning that children’s responses could be included in multiple categories for each question.

**“When you Go to the Doctor, What are the Kinds of Things that Make you Feel Worried or Scared?”**	**Frequencies**	**Illustrative Examples**
Needle procedures	95	“Needles, that’s it” “Taking my blood”
Medicine/Medical procedures/Tools (not needles)	35	“When they check my throat I might choke” “All the scary tools” “I might have to take medicine”
Pain	22	“The throat swabs really hurt my throat” “Because it always hurts at the doctor’s office”
Feeling uninformed/The unknown	20	“I didn’t know what was happening” “It was my first time so I didn’t know” “When an unexpected things happen it’s scary”
Illness and injury	19	“Worried that something is wrong” “What if I’m going to die”
No fears	16	“I’m not scared or worried about anything”
Medical personnel	3	“There was a different doctor, and I liked my old doctor”
Separation from parents	2	“If my mom and dad aren’t there”
Other response	22	“I bled a lot” “I was bored”
No response	8	“I don’t remember” “I don’t know”
**“How Can Someone Tell If you are Worried or Scared at the Doctor’s Office?”**	**Frequencies**	**Illustrative Examples**
Facial expression	66	“I would have a frown” “My face would turn red” “The look in my eye”
Verbal expression	55	“I would tell them I was scared”
Body actions	33	“I would be shaking” “I would move around a lot” “Sometimes I would breath really heavy”
Avoidance	27	“I would ask them off-topic questions” “I would hide underneath the car and not go in” “I’d ask to go home instead”
Vocal affect expression	15	“I would be screaming” “If I was crying”
Will not demonstrate fear	9	“I would keep it inside” “I would not show it”
Seek physical comfort from parent	5	“I would hold my mom’s hand” “I would sit on my parent’s lap”
Other response	11	“I have different ways of showing my feelings”
No response	13	“I don’t remember” “I don’t know”
**“What are Some Things you Wish Would be Different or be Changed the Next Time you Go to the Doctor’s Office?”**	**Frequencies**	**Illustrative Examples**
No needle procedures	54	“No more needles” “Needles wouldn’t be so sharp”
Reduced wait times	28	“I would not want to wait so long”
Medicine/Medical procedures/Tools (not needles)	27	“The blood pressure thing wouldn’t be so tight” “When they run lots of test and put too many things on me”
Reduced pain	25	“There wouldn’t be stuff that hurt”
No changes requested	20	“I would not want to change anything”
Improved décor in office/exam room	19	“I would make the walls look nicer for kids”
Activities available while waiting	13	“I would want more toys for kids my age”
Using medicine instead of a needle	9	“Instead of the needle, I could take medicine, like a pill”
Improved communication between doctor and patient	6	“Doctors would ask me more questions about how I feel” “I wish I could get the doctor to say words that I understand”
Other response	17	“All the sick people wouldn’t have to sit together”
No response	19	“I don’t remember” “I don’t know”

**Table 2:** Frequencies and illustrative examples from each category of responses to questions regarding children’s (*n* = 167) positive experiences during physician visits. The categories are not mutually exclusive, meaning that children’s responses could be included in multiple categories for each question.

“What are Some Things that you Liked or Made you Feel Happy When you Went to the Doctor? What are Some Things you Wish Would Stay the Same?”	Frequencies	Illustrative Examples
Rewards	53	“I get ice cream after” “I would like three stickers” “I get a new app on my iPad”
Being in better health	27	“I get medicine that makes me better” “I know I’m healthy when I leave”
Support from my healthcare provider	23	“My doctor is funny” “He was nice and he welcomed us”
Medicine/Medical procedures/Tools (not needles)	21	“I like getting my heart checked” “I get to ask about all the tools”
Receiving information about their personal health	16	“I found out what was happening, and what I had to do to get better” “I am happy to know nothing was wrong with me” “When I had strep throat, the doctor told me what it means”
Activities while waiting	16	“I like the TV in the waiting room” “I played with my dad and sister while we were waiting” “I liked playing with the toys in the waiting room”
When the visit is over	15	“Going home after” “When everything is done and I get to leave”
Support from parent	13	“My dad comforted me when I got my wart removed” “My mom and dad were with me, so I wasn’t scared”
Décor of the office/exam room	13	“The view from the window of the office” “I liked seeing the fishes” “There was Star Wars stuff on the walls and I liked looking at it”
Nothing positive about visits	11	“I don’t like anything”
Other response	6	“The seats were comfortable” “I liked everything”
No response	3	“I don’t remember” “I don’t know”

**Table 3:** Frequencies and illustrative examples from each category of responses to questions regarding children’s (*n* = 167) preferences for more participation and information during physician visits. The categories are not mutually exclusive, meaning that children’s responses could be included in multiple categories for each question.

“What are Some THINGS you Would Like your Doctor to Tell you More About During your Next Visit?”	Frequencies	Illustrative Examples
General/Unspecified information	34	“I just want to know what is going to happen” “They only tell my parents and not me, so I try to listen” “I want to know exactly what’s going on”
Medicine/Medical procedures/Tools (not needles)	31	“Do I need medicine? What’s inside the medicine?” “Maybe how the tool works and what it is called”
Needle procedures	30	“Are they going to give me a needle or something?” “If I’m getting a needle, they should tell me”
Information about their personal health	22	“She will never tell me if I’m sick or something” “I want to know how healthy I am” “If there is something wrong, is my body working?”
General appointment logistics	15	“How long will each part take, and when will the appointment be over?” “Will I have to come back another time?” “Maybe what’s going to happen”
No information	14	“I don’t want any more information” “I wouldn’t want to know anything”
Pain	6	“I want them to tell me about stuff that will hurt” “Tell me if it’s going to hurt today, or if the appointment will be calm”
Other response	11	“I don’t want them to tell me anything” “I don’t want them to tell me if I am getting a needle” “If they tell me what’s wrong with me, it freaks me out”
No response	40	“I don’t remember” “I don’t know”

## Data Availability

The data presented in this study are available on request from the corresponding author. The data are not publicly available due to privacy concerns.
